# Medicinal Potential of *Garcinia* Species and Their Compounds

**DOI:** 10.3390/molecules25194513

**Published:** 2020-10-01

**Authors:** Bruna Larissa Spontoni do Espirito Santo, Lidiani Figueiredo Santana, Wilson Hino Kato Junior, Felipe de Oliveira de Araújo, Danielle Bogo, Karine de Cássia Freitas, Rita de Cássia Avellaneda Guimarães, Priscila Aiko Hiane, Arnildo Pott, Wander Fernando de Oliveira Filiú, Marcel Arakaki Asato, Patrícia de Oliveira Figueiredo, Paulo Roberto Haidamus de Oliveira Bastos

**Affiliations:** 1 Graduate Program in Health and Development in the Central-West Region of Brazil, Federal University of Mato Grosso do Sul-UFMS, 79070-900 Campo Grande, Brazil; bruna.spontoni@gmail.com (B.L.S.d.E.S.); lidi_lfs@hotmail.com (L.F.S.); daniellebogo@hotmail.com (D.B.); rita.guimaraes@ufms.br (R.d.C.A.G.); priscila.hiane@ufms.br (P.A.H.); phaidamus43@gmail.com (P.R.H.d.O.B.); 2 Graduate of Pharmaceutical Sciences, Federal University of Mato Grosso do Sul-UFMS, 79070-900 Campo Grande, Brazil; hinokato@gmail.com; 3 Graduate of Electrical Engineering, Federal University of Mato Grosso do Sul-UFMS, 79070-900 Campo Grande, Brazil; felipe@nexsolar.com.br; 4 Laboratory of Botany, Institute of Biosciences, Federal University of Mato Grosso do Sul, 79070-900 Campo Grande, Brazil; arnildo.pott@gmail.com; 5 Faculty of Pharmaceutical Sciences, Food and Nutrition, Federal University of Mato Grosso do Sul-UFMS, 79070-900 Campo Grande, Brazil; wander.filiu@gmail.com; 6 Medical School, Federal University of Mato Grosso do Sul, 79070-900 Campo Grande, Brazil; marcel_arakakiasato@hotmail.com; 7 Laboratory PRONABio (Bioactive Natural Products)-Chemistry Institute, Federal University of Mato Grosso do Sul-UFMS, 79074-460 Campo Grande, Brazil; patricia.figueiredo@ufms.br

**Keywords:** Clusiaceae, phytochemical compounds, therapeutic effects

## Abstract

*Garcinia* is a genus of Clusiaceae, distributed throughout tropical Asia, Africa, New Caledonia, Polynesia, and Brazil. *Garcinia* plants contain a broad range of biologically active metabolites which, in the last few decades, have received considerable attention due to the chemical compositions of their extracts, with compounds which have been shown to have beneficial effects in several diseases. Our work had the objective of reviewing the benefits of five *Garcinia* species (*G. brasiliensis*, *G. gardneriana*, *G. pedunculata*, *G. cambogia*, and *G. mangstana*). These species provide a rich natural source of bioactive compounds with relevant therapeutic properties and anti-inflammatory effects, such as for the treatment of skin disorders, wounds, pain, and infections, having demonstrated antinociceptive, antioxidant, antitumoral, antifungal, anticancer, antihistaminic, antiulcerogenic, antimicrobial, antiviral, vasodilator, hypolipidemic, hepatoprotective, nephroprotective, and cardioprotective properties. This demonstrates the relevance of the genus as a rich source of compounds with valuable therapeutic properties, with potential use in the prevention and treatment of nontransmissible chronic diseases.

## 1. Introduction

Research into medicinal plants can provide essential knowledge about drugs from plants and for the production of phytotherapeutic agents. Understanding the chemical compositions of herbs is a necessary step in obtaining standards for their quality specifications, using both analytical and phytochemical determinations. Thus, materials destined for medicinal purposes must be submitted to a protocol of evaluation for their quality standards, applying all possible means of botanical and chemical analyses before commercialization [1].

The nutraceutical properties of medicinal plants can be determined by their carbohydrates, proteins, vitamins, minerals, and metabolites, such as flavonoids and antioxidants. Secondary metabolites, such as phenols and flavonoids, also contribute considerably to their medicinal functions. Fruits also have medicinal properties, their most relevant secondary metabolites being phenols and flavonoids [2].

Among medicinal plants, the former family *Guttiferae*, comprising circa 140 genera and 1200 species [3] (which was split into various families), and *Clusiaceae*, with 14 genera and 600 species, stand out. *Garcinia* (=*Rheedia*) is a plant genus of *Clusiaceae*, distributed throughout tropical Asia, Africa, New Caledonia, Polynesia, and Brazil. Species of *Garcinia* are rich and valuable sources of bioactive compounds with relevant therapeutic properties, such as anti-inflammatory and analgesic properties [4,5,6,7,8,9]. A great variety of compounds, mainly polyisoprenylated benzophenones, flavonoids, and xanthones have been isolated from *Clusiaceae* species. Thus, species of the genus *Garcinia* have proved to be rich sources of compounds with relevant therapeutical properties [7,8,10,11]. *Garcinia* species are rich in secondary metabolites, such as prenylated and oxygenated xanthones [11] with biological activities such as antifungal [12], anti-inflammatory [13], antitumoral [14], antioxidant [15,16], Human Immunodeficiency Virus (HIV)-inhibitory [7], and antilipidemic properties [14,17].

The genus *Garcinia* contains a broad range of biologically active metabolites, and these, in the last few decades, has received considerable attention for the chemical composition of their extracts, being rich in derivates of polyisoprenylated benzophenones, polyphenols, bioflavonoids, and xanthones [18,19,20].

In traditional medicine, the fruits of *Garcinia* have been utilized in infusions for treating wounds, ulcers, and dysentery [20]. Extracts of the pericarp, epicarp, and seeds of *Garcinia* have demonstrated antioxidant, anti-inflammatory, leishmanicidal, and antiprotozoal activities [21,22,23]. Another study also reported the presence of the bioflavonoids volkensiflavone, fukugetin [24], and prenylated xanthones [25]. These compounds have been associated with biological activities such as free-radical scavenging, antiulcer effects [26], cytotoxicity, inhibition of nitric oxide synthase [27], chemoprevention of cancer [28], induction of apoptosis [29], anti-HIV [30], and trypanocidal effects [31].

Some metabolites isolated from the genus *Garcinia* have already shown anticancer activities. Garcinol, a polyisoprenylated benzophenone obtained from *Garcinia*, was evaluated in vitro and in vivo, and induced apoptosis and arrest of the cellular cycle, inhibition of angiogenesis, and modulation of the gene expression of carcinogenic cells [32]. Xanthones found in *Garcinia* species have demonstrated effects against human cervical cancer, lung cancer cells, and hepatocellular carcinomas [33,34].

Some biflavonoids derived from *Garcinia* have also been evaluated for various activities, including chemoprevention properties. Among these, kolaviron has been pointed out, which presents the capacity to eliminate free radicals, inhibit proteins related to the stress response, and interfere with the DNA-binding activities of some transcription factors [35], as well as showing inhibitory activity against aromatase [36], the enzyme which catalyses the final step of the biosynthesis of estrogen, considered a key target for the development of drugs against estrogen-dependent breast cancers [37].

Given the presence of various compounds with several functions in these organisms, our work had the objective of reviewing the benefits presented by five species of *Garcinia* (*G. brasiliensis*, *G. gardneriana*, *G. pedunculata*, *G. cambogia*, and *G. mangstana*).

## 2. *Garcinia* Species and Bioactive Compounds

### 2.1. Garcinia Brasiliensis

*Garcinia brasiliensis* Mart. (*Rheedia brasiliensis* (Mart.) Planch. & Triana) is a species native to the Amazonian region, which is cultivated all over Brazil and which is commonly known as “bacuri”, “bacupari”, “porocó”, “bacuripari”, and, in Bolivia, “guapomo”. This tree has yellow fruit with mucilaginous, white, and edible sour-sweet pulp, which is utilized by the local people for its anti-inflammatory [22,38], antinociceptive [22], antioxidant, and antitumoral [39] properties. In some countries, such as Thailand, Sri Lanka, Malasia, the Philipines, and India, the ripe fruits are used in traditional medicine to treat abdominal pain, diarrhea, dysentery, infected wounds, suppuration, and chronic ulcers [11].

Some compounds found in the fruit peel are oxygenated sesquiterpenes—volatile oils obtained by hydrodistillation—presenting γ-muurolene (**1**; 10.3%), spathulenol (**2**; 8.7%), δ-cadinene (**3**; 8.3%), torreiol (**4**; 8.0%), α-cadinol (**5**; 7.0%), cadalene (**6**; 6.3%), and γ-cadinene (**7**; 5.3%) [31]. When tested, the essential oil presented anti-inflammatory activity at a dose of 100 mg/kg [22,31].

The ethanolic extract of *G. brasiliensis* leaves at concentrations of 30 and 300 mg/kg demonstrated anti-inflammatory action in rats and antinociceptive action in mice, corroborating the traditional use of species of *Garcinia* against inflammation of the urinary tract and inflammatory pains such as arthrosis. The biflavonoids procyanidin (**8**), fukugetin (**9**), amentoflavone (**10**), and podocarpusflavone A (**11**), isolated from *G. brasiliensis*, represent a therapeutic strategy to control diseases related to oxidative stress, controlling inflammation and reducing the harmful effects of reactive species of oxygen (ROSs). Furthermore, biflavonoids have exhibited potent inhibition of the oxidative hemolysis and lipidic peroxidation induced by 2,2′-azobis amidinopropane (AAPH) in human erythrocytes, demonstrating the anti-inflammatory and antioxidant properties of the compounds present in *G. brasiliensis* [40].

Another effect presented by the species is leishmanicidal activity [21,41]. The leishmanicidal activities of the hexane extract and ethyland ethanolic acetate at 5.0 mg/mL were evaluated, as well as those of molecules obtained from the extraction of the pericarp of *G. brasiliensis* in an in vitro model. The hexane extract presented the best activity on extracellular (promastigote) and intracellular (amastigote) forms of *Leishmania* (*L*.) *amazonensis*, compared with other extracts. Following those results, fractions of the most efficient extract were made, resulting in three purified prenylated benzophenones, 7-epi-clusianone (**12**), garciniaphenone (**13**), and guttiferone-a (**14**) [21,42]. These results suggested that the hexane extract and the polyprenylated benzophenones isolated from *G. brasiliensis* have relevant leishmanicidal activities and provide potential compounds for the development of new drugs against leishmaniasis. The compound found in the extract, morelloflavone-7,4′,7′′,3′′′,4′′′′-penta-*O*-acetyl (**15**), was prepared by acylation and alkylation reactions from the compound morelloflavone isolated from the ethyl acetate extract of *G. brasiliensis* fruits, which demonstrated leishmanicidal, antiproteolytic, and antioxidant activities, as well as low cytotoxicity in in vitro models, at a concentration of 400 μg/mL [41].

The compound 7-epiclusianone (**12**) found in the pericarp of *G. brasiliensis* fruits exhibited biological activity in vitro against trypomastigotes of *Trypanosoma cruzi* [9], and a potent vasodilatory effect on the endothelium [42]; antianaphylactic [43], anti-HIV [29], antimicrobial [5,44,45,46], antispasmodic [39], antiproliferative [45], and leishmanicidal activities, have also been attributed to this benzophenone [21].

A study evaluated the analgesic and anti-inflammatory effects of benzophenone 7-epiclusianone extracted from the epicarp of *G. brasiliensis* using experimental models of rats and mice [22]. In the test, benzophenone 7-epiclusianone (**12**) exerted an anti-inflammatory effect, which was verified through the reduction of mouse paw edema induced by carrageenin and the inhibition of recruitment of leucocytes to the peritoneal cavity, as well as the nociception induced by intraperitoneal injection of acetic acid. The substances associated with the extract components were capable of absorbing ultraviolet-B (UVB) radiation, preventing the induced inflammatory process. The absorption of UVB radiation by components of the ethanolic extract could impede the installation of oxidative stress and, consequently, lipidic peroxidation, antioxidant capacity, and removal of free radicals, contributing to a photoprotective effect [47].

Treatment with 7-epiclusianone (**12**) altered the cell-cycle progression; furthermore, the capacity to form cell colonies was significantly reduced, demonstrating long-term effects. This demonstrated that 7-epiclusianone (**12**) is a relevant natural benzophenone with antineoplastic activity in a model of glioblastoma—a tumor with chemoresistance, demonstrating influence on growing cells, cell-cycle dynamics, apoptosis, and ability to form colonies [48]. The 7-epiclusianone (**12**) was isolated from *G. brasiliensis* for the treatment of schistosomiasis, showing efficacy against *Schistosoma mansoni* adult worms, cercariae, and schistosomula in vitro [49].

Administration of the ethanolic extract to rats at a concentration of 300 mg/kg produced an increased antioxidant activity through the reduction of inflammation and adiposity in obese rats. The antiobesity effect of the treated group was related to the negative regulation of the lipogenic gene of the lipoprotein lipase (LPL), the proteins of Tumor Necrosis Factor Alpha (TNF-α) and Interleukin 1 (IL-1), diminishing adipogenesis, adipocyte size, and body weight, when compared with the control group [50].

The following components have been isolated from the epicarp of *G. brasiliensis* fruit: a new glycosylated biflavonone, morelloflavone-4′′′-*O*-β-d-glycosyl (**16**), and the known compounds 1,3,6,7-tetrahydroxyxanthone (norathyriol; **17**), morelloflavone (fukugetin; **9**), and morelloflavone-7′′-*O*-β-d-glycosyl (fukugesid; **18**). These compounds presented antioxidant activity after the isolation of natural biflavonoids from the plant [41].

The ethanolic extract of *G. brasiliensis*, at a concentration of 300 mg/kg, reduced oxidative stress and inflammation in obese rats with cardiac insufficiency, and presented a promising strategy for beneficial microbiota modulation. That demonstrates the potential protective effects of two phenolic compounds, morelloflavone and 7-epiclusianone (**12**), present in the extract [51].

**Figure 1:**
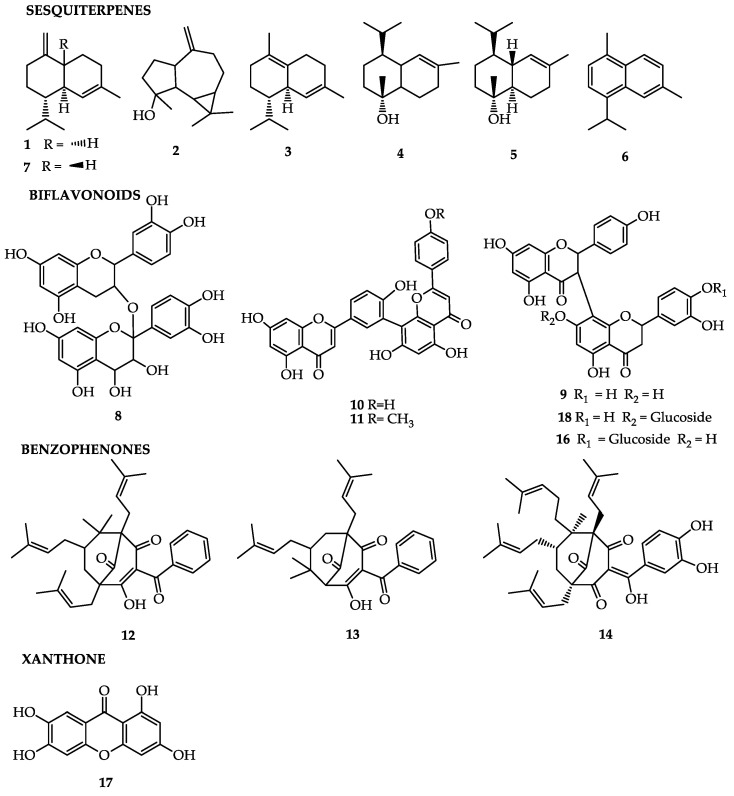
Bioactive compounds of *Garcinia brasiliensis*.

It is worth noting the method of extraction of the bioactive compounds. The use of the solvent N-hexane has demonstrated to be the most adequate for extracting guttiferone A and/or 7-epiclusianone, whereas the highest levels of fukugetin and norathyriol (**17**) were detected in the ethyl acetate fraction [37]. Table 1 and Figure 1 summarize the main compounds, plant part from which they were extracted, and their related activities.

### 2.2. Garcinia Gardneriana

*Garcinia gardneriana* (Planch. & Triana) Zappi (*Rheedia gardneriana* Planch. & Triana) (*Clusiaceae*) is native to the Atlantic forest and grows throughout Brazil. It is an easily cultivated fruit tree which is often found in domestic orchards. It is regionally known as “bacupari”, “bacopari”, “bacopari-miúdo”, or “mangostão-amarelo” [53]. The fruit is initially dark green, becoming yellowish-green or yellow-orangish when ripe. The fruit peel (or epicarp) is smooth and coriaceous. The pulp is white, edible, and sour-sweetish, formed by the mesocarp and endocarp [52,54]. A study on its fruits identified two phytosterols—sitosterol and stigmasterol—which have already presented anti-inflammatory and anticancer activities in other studies, with the isolation of these compounds achieved in fruits of the genus *Garcinia* [37,52]. Furthermore, four sesquiterpenes—α-copaene (**19**), α-muurolene (**20**), γ-cadinene (**7**), and cadinene (**21**)—were identified in the fruit peel, besides triterpene oleanolic acid (**22**) [52].

The plant has generally been applied for several purposes in folk medicine, such as inflammatory problems including skin disorders and wounds, as well as for the treatment of pain and infections [38]. The leaves, bark, and roots are the most utilized parts, typically prepared as infusions, decoctions, or macerates, either separately or combined with other natural products [38]. 

Evaluation of a hydroalcoholic extract of *G. gardneriana* revealed that it diminished the quantity of melanin in B16F10 melanoma cells and, specifically, promoted the inhibition of tyrosinase activity [55]. The ethanolic extract conferred an additional beneficial effect to the skin as the plant has a high content of bioflavonoids, which are considered to be able to reduce the potential oxidative damage produced in the skin after exposure to ultraviolet radiation [56]. *G. gardneriana* presented a potential source of bioactive compounds with a significant antiproliferative effect in breast neoplastic lines in animals [57].

*Garcinia gardneriana* is very rich in secondary metabolites. Some phytochemical analyses have identified xanthones, steroids, triterpenes, and flavonoids in different parts of the plant [14,41,42,43], which have been associated with pharmacological effects such as anti-inflammatory, antinociceptive, antibacterial, and antiparasitic activities [38,58,59,60].

Phytochemical analyses of *G. gardneriana* detected several classes of compounds, such as steroids, triterpenes, biflavonoids, and xanthones [61]. Several biflavonoids found and identified as volkensiflavone (**23**), 13-naringenin-II 8-eriodictyol (GB-2a; **24**), fukugetin (or morelloflavone; **9**), and fukugesid (**18**) have demonstrated analgesic effects [62]. A new biflavonoid isolated from *G. gardneriana* leaves, named GB2a-OMe (**25**), also presented a significant analgesic effect in the formalin test in mice in the neurogenic and inflammatory phases [63].

The compound GB-2a significantly inhibited the melanin content without reducing cell viability, suggesting its great potential for medical use as a hypopigmentation agent, for cosmetic and clinical applications related to skin clearing [64]. The compound fukugetin (or morelloflavone) showed an anti-inflammatory activity in mouse paw edema induced by carrageenin at a concentration of 300 mg/kg, rendering the plant a potential target for the development of new compounds to be explored as alternatives to drugs with anti-inflammatory activity that are already in use [65].

**Figure 2:**
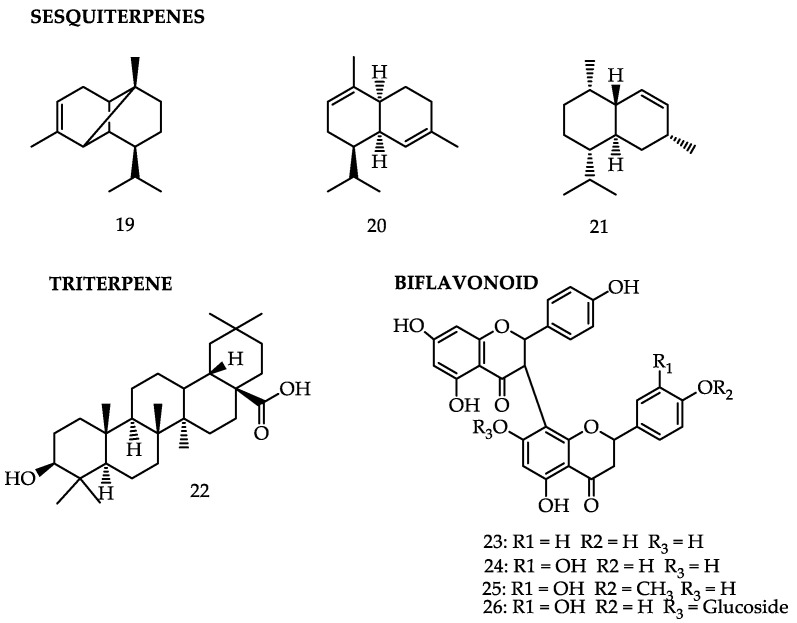
Bioactive compounds of *Garcinia gardneriana*.

The biflavonoids isolated from *G. gardneriana*, such as morelloflavone (**9**), Gb-2a (**24**), and Gb-2a-7-*O*-glucose (**26**) were submitted to an in vitro trial in order to evaluate their modulatory effects on aromatase, utilized for cancer treatment. The results showed that all biflavonoids were able to inhibit the enzyme, with IC_50_ values varying from 1.35 to 7.67 μM. This demonstrates that these biflavonoids are a relevant source of new aromatase inhibitors, with focus on the development of new anticancer agents. This reinforces that the species is an important source of bioactive compounds, with applications concentrated mainly in the treatment of estrogen-dependent breast cancers [65]. Table 2 and Figure 2 summarize the main compounds of *Garcinia gardneriana*, the plant parts from which they were extracted, and their related activities.

### 2.3. Garcinia Pedunculata

*Garcinia pedunculata* Roxb. (*Clusiaceae*) is a tree endemic to some Asian regions—namely to parts of Myanmar and oriental parts of India. The fruit is known as “taikor” in Bangladesh and “amlavetasa” in India [66]. It also is an indigenous medicinal plant. Traditionally, the fruit has been utilized by people to treat several gastrointestinal disorders [67], as a cardiotonic, and as an emollient. It is also utilized in the treatment of asthma, cough, bronchitis, diarrhea, and fever [68].

The fruit is greenish-yellow and is utilized as an ingredient in several meat dishes as a culinary adstringent [69]. The fruit of *G. pedunculata* contains 7.93% carbohydrates, 0.95% reducing sugars, 4.93% total proteins, and 0.20% total fats. Regarding the composition of vitamins and minerals, it has 2.48 mg/100 g sodium, 27.3 mg/100 g potassium, 13.21 mg/100 g calcium, 35.43 mg/100 g magnesium, 10.12 mg/100 g iron, 4.32 mg/100 g phosphorus, 49 µg/100 g thiamine, 276 µg/100 g riboflavin, 47 µg/100 g niacin, 35.43 µg/100 g ascorbic acid, and 8.12 µg/100 g vitamin B12 [2]. 

Phytochemical studies have shown that the dry fruits contain hydroxylcitric acid, benzophenones, garcinol, pedunculol, and isogarcinol (cambogin), the first having been reported as possessing antioxidant activity [16], and the second and third with anticancer, anti-inflammatory, and antiparasitic activities [24,70]. Dry fruits have been selected for different actions and have shown anti-inflammatory, hepatoprotective, cardioprotective, and antioxidant pharmacological activities in vitro [71,72]. Phytochemical analyses have revealed the presence of phytochemicals such as pedunculol (**27**), garcinol (**28**), cambogin (**29**) [73], and (α)-hydroxylcitric acid (**30**) [70]. Hexane and chloroform extracts of *Garcinia pedunculata* showed antioxidant activity, helping in the elimination of free radicals and showing strong antimutagenicity, the hexane extract being more reactive than that of the chloroform extract [73].

Among the reported benefits of *G. pedunculata* fruit are antioxidant [70,71,72,73,74,75], antimicrobial [76], anti-inflammatory [71], hypolipidemic [77], hepatoprotective [66], and nephroprotective effects [71], as well as cardioprotective properties [77]. The peel and the pericarp of dry fruits have been shown to contain benzophenones, pedunculol (**27**), garcinol (**28**), cambogin (**29**), and hydroxycitric acid (HCA; **30**) [70], some of which are potent antioxidants. Some research has suggested that benzophenones and garcinol present protective effects against the toxicity of carbon tetrachloride in hepatocytes of rats [70] and anti-inflammatory effects in hepatocytes of mice [78]. An ethanolic extract of the fruit showed significant hepatoprotective, cardioprotective, and hypoglycemic activities in the treatment of Long Evans rats with a daily dose of 1000 mg/kg for 21 days [79]. The nephroprotective effect detected with the administration of a water extract of the fruit peel at concentrations of 200 and 400 mg/kg of weight was attributed to its general cytoprotective effect, which promptly impeded the ischemic damage caused by acute toxicity by cisplatin, a cytotoxic agent that has effects on the kidneys, liver, and neural tissues [80].

Administration of the extract of *G. pedunculata* fruit significantly reduced blood glucose levels, demonstrating the possibility of reduction of hyperglycemia, diabetes, diabetic comorbidities, and protection against damages induced by oxidative stress [81]. Administration of methanolic extract at a concentration of 200 mg/kg attenuated hyperlipidemia and oxidative stress in the studied animals [77]. Evaluation of a methanolic extract of the fruit showed antioxidant activity, having free-radical scavengers and the capacity to protect cells from lipidic peroxidation, which is associated with the treatment of degenerative diseases and diabetes [77,82].

A recent study on an aqueous extract of fruits of *G. pedunculata* given to rats at 200 and 400 mg/kg of body weight observed a significant reduction in damage caused by colitis, preventing oxidative peroxidation. At the dose of 400 mg/kg, the lipidic peroxidation was reverted significantly, and in several parameters of inflammation generated in the colon showed improvement (i.e., the punctuation of macroscopic damage, lipidic peroxidation, and histopathological exam of the colon tissue), demonstrating its therapeutical potential for the treatment of colitis [83].

Analysis of pericarp and peel separately reported a diversity of xanthones in the form of the compounds peduxanthone-d (**31**), -E (**32**), and –F (**33**), standing out in the pericarp [33], which have shown anticancer activity [65]; meanwhile, garbogiol (**34**), present in the peel [33], has been reported as an inhibitor of α-glucosidase [33].

**Figure 3:**
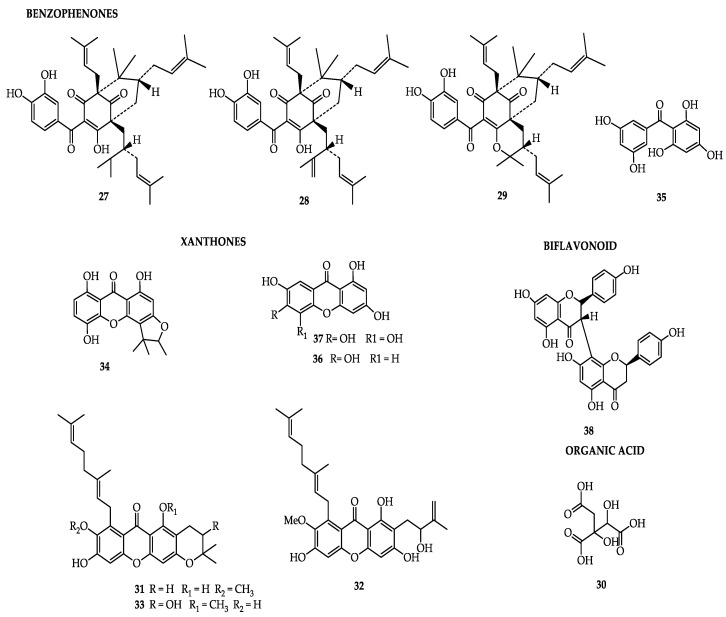
Bioactive compounds of *Garcinia pedunculata*.

Besides the fruits, a study on the heartwood of the species [19] identified benzophenone2,4,6,3′,5′-pentahydroxybenzophenone (**35**) and the xanthones 1,3,6,7-tetrahydroxyxanthone (**36**) and 1,3,5,7-tetrahydroxyxanthone (**37**) to have antioxidant activity [42] and LDL-c-oxidation-inhibitory activity, respectively; additionally, the biflavonoids GB-1a (**38**) and volkensiflavone (**23**) have shown antioxidant activity [84] and antitumoral activity [74], respectively. Table 3 and Figure 3 summarize the main compounds, the plant parts they have been extracted from, and their related activities.

### 2.4. Garcinia Cambogia

*Garcinia cambogia* L., known as Malabar tamarind, is a plant native to Southeast Asia. The fruit is used as a food preservative, carminative, and flavoring agent [82]. The fruit contains hydroxycitric acid (HCA; **30**) and is a popular ingredient utilized for weight reduction [85,86]. Semwal [85] presented a revision of the species, citing the presence of organic acids, such as HCA, in the fruits, as well as the xanthones oxy-guttiferone-I (**40**), -K (**41**), -K2 (**42**), and -M(**43**), and the benzophenones guttiferone-I (**44**), -J (45), -K (**46**), -N(**47**), and -M (**48**). Guttiferone-K (**46**) and guttiferone-M (**48**) are inhibitors of topoisomerase II [87]. In that same study, the presence of the xanthone garbogiol was reported in the roots. In the peel, the presence of rheediaxanthone-A [86], benzophenonesgarcinol (**28**), and isogarcinol (**29**) was also reported.

In Indian medicine, the extract of *G. cambogia* is used to treat ulcers, hemorrhoids, diarrhea, dysentery, and some types of cancer, such as leukemia [88]. Initial studies on seeds confirmed that they have antifungal [89], anticancer [28,90], antihistaminic [91], antiulcerogenic [92], antimicrobial [93], antiviral [94], and vasodilatory effects [95]. The gastroprotective effects seem to be related to its capacity to diminish acidity and increase the mucosal defenses [92,96]. Furthermore, the extract presented hypolipidemic [95], antiadipogenic, and appetite-suppression effects in experimental animals through the inhibition of the expression of the early adipogenic transcription factor CCAAT enhancer-binding protein alpha (C/EBP alpha), which regulates adipogenesis [97,98,99].

The hypolipidemic effect of the *G. cambogia* extract has been attributed to its high content of flavonoids [100]. The generated anti-inflammatory effects resulted in the improvement of some parameters analyzed in experimental colitis, where 2,4,6-Trinitrobenzenesulfonic acid (TNBS)/ethanol caused lesions characterized by severe necrosis of the mucosa, hyperemia, and focal adhesion to adjacent organs. Administration of the extract by oral gavage at a dose of 1.0 g/kg reduced the length of the lesions observed macroscopically; thus, it may provide a source to search for new anti-inflammatory compounds useful in the treatment of intestinal inflammatory diseases [101].

*Garcinia cambogia* showed an antiobesity effect and a significant reduction in the values of triacylglycerol (TAG) of the adipose tissue and liver of the tested groups; however, it significantly increased the TAG pool of the gastrointestinal system [95,102,103]. The plant also reduced the serum levels of total cholesterol, triglycerides, and insulin, as well as the intolerance of glucose and levels of alpha-TNF associated with hyperlipidic diets [95,102,103,104,105]. *G. cambogia* extracts have also been shown to trigger the myotubes and skeleton cells to absorb glucose and to equilibrate the glucose levels in the blood [106].

This species also has already shown favorable results in tests in humans—either healthy or bearing some nontransmissible chronic disease—for 6 months. Treatment with 500 mg of HCA, twice a day, promoted weight loss and reduction of fatty mass, visceral fat, total cholesterol, and glycemic profile. Furthermore, an increase of the basal metabolic rate was perceived, independent of sex, age, or bearing hypertension, diabetes mellitus type 2, or dyslipidemia [107].

The HCA (**30**) present in *G. cambogia* is a potent and competent inhibitor of adenosinetriphosphate (ATP) citrate lyase, which is a key enzyme in the synthesis of fatty acids, cholesterol, and triglycerides [85,108]. It also regulates the level of serotonin, which has been associated with satiety, increased oxidation of fat, and decreased gluconeogenesis [85,109]. This explains how the compound exerts weight-loss activity, with reduced food ingestion and reduction of accumulated gain of body fat [85,108,109,110]. HCA (**30**) presents a chemical structure similar to citric acid and, therefore, inhibits the action of adenosine triphosphate (ATP) citrate lyase in the citric acid cycle. This action inhibits the conversion of citric acidinacetyl-coenzyme A (CoA) and suppresses the synthesis of fatty acids. The increased quantity of citric acid that is not converted into acetyl-CoA leads to acceleration of the production of glycogen from glucose. Thus, the ingestion of HCA (**30**) stabilizes glucose levels in the blood, resulting in the suppression of feelings of hunger. Therefore, it is also expected to show a preventive effect against hyperphagia [111,112,113,114,115]. Earlier studies showed that HCA (**30**) reduced the build-up of lipidic droplets and accelerated the energy metabolism, besides protecting cells from oxidative stress, as well as increasing the antioxidant status and mitochondrial functions [116,117].

Despite the benefits present in the species, some studies have shown that its consumption can cause adverse effects, such as headache, dizziness, dry mouth, nausea, and diarrhea [118]. Recent studies have described (hypo)mania and/or psychosis after the consumption of *G. cambogia* [87,119,120,121]. Some liver complications have also been reported, such as hepatotoxicity, with acute hepatic lesions, acute hepatitis, and hepatic insufficiency requiring transplant [122,123,124,125]. The complications from *G. cambogia* include mania or hypomania, mania with psychosis, and serotonin syndrome [10,126]. When taken over the recommended dose, individuals should be aware that the extract of *G. cambogia* can also lead to ocular complications [127].

HCA (**30**), the main active ingredient of *G. cambogia* extracts, presents effects of inhibiting the recapture of serotonin, inhibiting acetylcholinesterase, increasing the oxidation of fatty acids, and reducing lipogenesis [85]. The serotoninergic effects of HCA (**30**) are worrisome and can contribute to serotonin syndrome when combined with serotonin recapture inhibitors [109].

**Figure 4:**
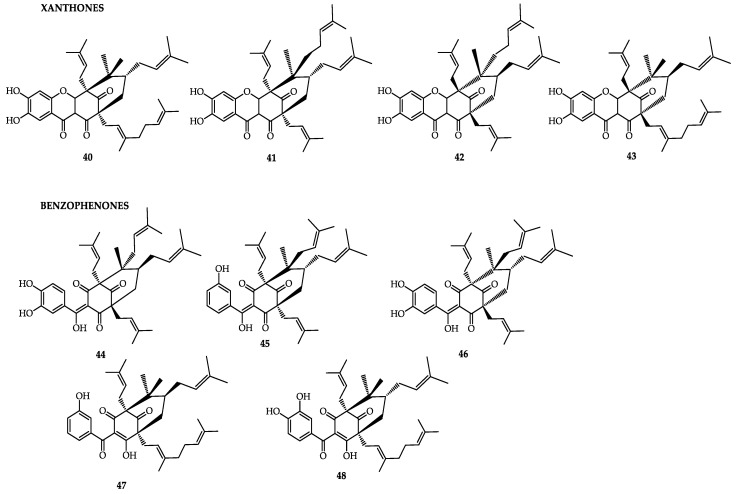
Bioactive compounds of *Garcinia cambogia*.

Some cases have been reported of acute pancreatitis secondary to the use of *G. cambogia* [128,129]. The pathogenesis of how such an increased risk of acute pancreatitis may occur is not clear; however, there is evidence that active oxygen species may play a central role in this pathogenesis. *Garcinia cambogia* increases lipidic peroxidation and positively regulates the expression of superoxide dismutase and glutathione peroxidase messenger ribonucleic acid (RNA) [130]. Lipidic peroxidation also increases oxidative stresss and can increase the risk of acute pancreatitis in patients using the species [131]. *G. cambogia* can cause other severe adverse events, including hepatoxicity and secondary acute hepatic insufficiency [124,132]. Other studies have also shown acute necrotizing eosinophilic myocarditis, rhabdomyolysis, serotonin toxicity, and nephropathy secondary to the use of *G. cambogia* [87,121,122,128,129,130,131,132,133,134,135,136]. Table 4 and Figure 4 describe the main compounds, the plant parts they have been extracted from, and their related activities.

### 2.5. Garcinia Mangostana

*Garcinia mangostana* L. is a tropical evergreen fruit tree native to Southeast Asia, with the popular name of mangosteen, known for containing several constituents including xanthones, flavonoids, triterpenoids, and benzophenones [64]. In many Asian countries, the peel of *G. mangostana* has been used in traditional medicine to cure various diseases, such as diarrhea, dysentery, skin infections, mycosis, inflammation, cholera, and fever [139,140]. Fruit extracts have exhibited antioxidant [141,142], anti-inflammatory [143,144], antibacterial [145], and antidepressive effects [146]. In particular, α-mangostin (AM; **49**), a primary component of *G. mangostana*, has presented substantial pharmacological properties [147,148], including antioxidant activity in the treatment of age-related macular degeneration and protecting the retina from light damage [149].

Its pharmacological properties have been attributed to the presence of polyphenols such as xanthones, anthocyanins, phenolic acids, and flavonoids [142,150]. It has demonstrated antioxidant, anti-inflammatory, antitumoral, antibacterial, antifungal, antiviral, and anti-allergic properties [150,151]. Alfa-mangostin (**49**) is one of the most abundant xanthones in *G. mangostana*. The presented anti-inflammatory effects have been evidenced by reduced levels of TNF-α and IL-6 [152,153]. It has also shown antihyperglycemic, antioxidant, and anti-inflammatory effects, as well as improved blood flux and integrity of the retina [153,154]. The fruit has also produced improved results in terms of adiposity, hyperlipidemia, insulin resistance, and hepatic lesion related to ageing [155].

Mangosteen is used, in the form of an infusion, as a tonic for fatigue and as a digestive [139]. It can also be utilized for its medicinal properties in hemorrhoids, flood allergies, arthritis, tuberculosis, mycosis, mouth sores, fever, candidiasis, abdominal pain, suppuration, leucorrhea, and convulsions [140].

Some studies have shown the antihyperglycemic power and antidiabetic activity of mangosteen. Mangosteen pericarp extract has shown efficacy in the reduction of cholesterol levels and lipidic peroxidation, besides improving the kidney structure and function in fastening diabetic rats [156,157]. The hypoglycemic power is due to the inhibition of the activity of α-glucosidase and α-amylase: the enzymes responsible for the digestion of carbohydrates [158]. The xanthones mangostaxanthone-I (**50**), -II (**51**), and -VIIII (**52**), found in the pericarp, have been reported as inhibitors of the activity of α-amylase [133]; meanwhile, mangostenone-F (**53**), gartanin (**54**), α-magostin (**49**), and γ-magostin (**55**) have been shown to be inhibitors of the activity of α-glucosidase. Besides these compounds, the presence of the xanthones β-magostin (**56**), 3-isomangostin (**57**), magostenone-C (**58**) and -d (**59**), as well as the flavonoids aromadendrin-8-C-β-*d*-glucopyranoside (**60**) and epicatechin (**61**), in the fruits corroborates those studies, which have presented hypoglycemic and antiobesity activities [134].

A hepatoprotective effect, which has previously been shown as one of the actions of α-mangostin [159], and renoprotective action were also found in streptozotocin-induced diabetic mice [160]. Some authors have cited the compound α-mangostin (**49**) as having anticancer activities, being capable of inducing cell death via apoptosis of human colorectal carcinomas [161,162]. This compound has presented antioxidant activity and evidences the benefits of the fruit in improving the kidney structure and function in diabetic rats [157]. In human melanoma, breast cancer, and epidermoid carcinoma, the compound α-mangostin had a cytotoxic effect, inducing the death of the cited cells [163,164].

One study on the mangosteen pericarp demonstrated a wide range of activities, including antifungal, antioxidant, antiobesity, and antidiabetic properties [139]. Its hypoglycemic power is due to the inhibition of the activity of α-glucosidase and α-amylase, enzymes responsible for the digestion of carbohydrates [158].

Some studies have presented satisfactory results with respect to the endogenous antioxidant system, demonstrating a high level of antioxidant enzymes in the organisms of the tested animals. Such effects suggest the capacity of the fruit to eliminate free radicals from the biological system [165]. Human adipocytes treated with α-mangostin (**49**) showed a decrease in the expression of inflammatory genes, as well as reducing insulin resistance [166]. Indeed, the daily consumption of a mangosteen drink for 30 days in healthy adults resulted in reduction of the inflammatory markers and increased the antioxidant capacity of human blood, due to reduction of the inflammatory marker C-reactive protein, reducing the risk of inflammation and chronic diseases related to immunity [167]. Thus, it has been proven that the mangosteen is a plant which can provide benefits in the development of drugs for the prevention and treatment of numerous diseases, mainly as it is a rich source of xanthones and other bioactive substances [159].

**Figure 5:**
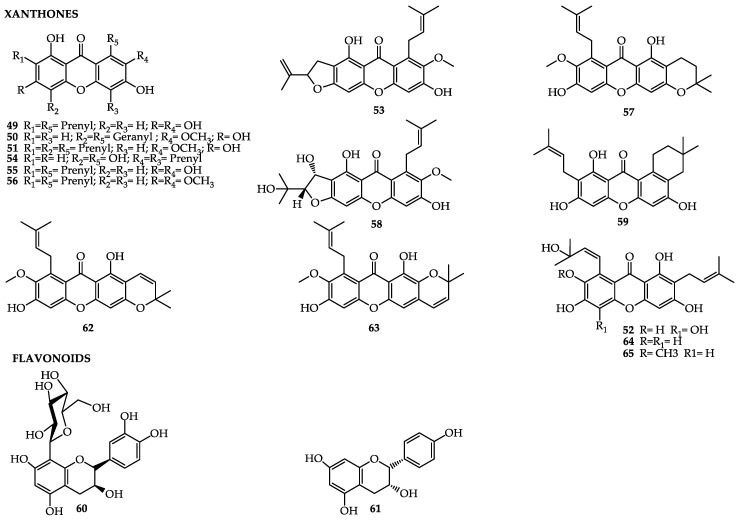
Bioactive compounds of *Garcinia mangostana*.

A study on rats fed daily with an aqueous extract of mangosteen pericarp (100 and 200 mg/kg, 38 days) showed that they exhibited significant improvements in memory loss. The extract, rich in xanthones, was also capable of restoring acetylcholinesterase activity in the dysfunction induced by lead in red blood cells and brain tissue. The presence of the xanthones α- and γ-mangostin (**55**), 3-isomangostin (**57**), gartanin (**54**), garciniafuran (**62**), 9-hydroxycalabaxanthone (**63**), and garcinone -C (**64**) and -d (**65**) was verified [134]. Table 5 and Figure 5 list the main compounds of *Garcinia mangostana*, the plant part where they have been extracted from, and their related ativities.

## 3. Conclusions

Plant species of the genus *Garcinia* are a relevant source of bioactive compounds. This review compiled the bioactive compounds found in five species of the genus *Garcinia*, as well as the effects of several types of extracts of different plant parts. Plants from genus *Garcinia* exhibits healing properties with anti-inflammatory effects, for the treatment of such ailments as skin disorders, wounds, pain, and infections, as well as presenting antinociceptive, antioxidant, antitumoral, antifungal, anticancer, antihistaminic, antiulcerogenic, antimicrobial, antiviral, vasodilatory, hypolipemic, hepatoprotective, nephroprotective, and cardioprotective properties. It was possible to observe that, across all the species mentioned in the present review, most of the studies carried out were in vitro experiments. Some tests have already been started in in vivo models; however, these are recent studies evaluating the effectiveness of the plant in treating diseases in animal models. These studies are promising and open up new perspectives on the use of the compounds present in these species, offering new perspectives on the possibility of developing new drugs. For this to be effective, it is necessary to initiate plant-use tests in humans, in order to analyze their effectiveness in treating diseases. Therefore, considering the high number of compounds found in plants of the genus and their beneficial effects, additional studies are required to support the development of new products with therapeutic properties for the prevention and treatment of various diseases; most importantly, non-transmissible chronic diseases. Therefore, these plants provide a promising potential source of natural biomolecules for pharmaceutical and medicinal applications.

## Figures and Tables

**Table 1:** Compounds present in different parts of *Garcinia brasiliensis* and their related activities.

*Garcinia Brasiliensis*		
Sesquiterpenes		
Compounds	Plant Part	Activity
α-Ylangene; α-Copaene; β-Bourbonene; β-Elemene; β-Caryophyllene; β-Gurjunene; Aromadendrene; α-Humulene; Drima-7,9(**11**)-diene; y-Muurolene-10; Germacrene D; β-Selinene; Viridiflorene; α-Muurolene; γ-Cadinene; cis-Calamenene; Cadina-1,4-diene; α-Cadinene; α-Calacorene; Longicamphenylone; Ledol; Spathulenol; Globulol; Salvial-4(**14**)-en-1-one; Guaiol; Viridiflorol; Humuleneepoxide II; 1,10-Diepicubenol; 1-Epicubenol; Cubenol; Cedr-8(**15**)-en-9a–ol; Torreyol; Selin-11-en-4a-ol; α-Cadinol; Khusinol; Cadalene; 14-Oxy-α-muurolene.	Peel [31]	Anti-inflammatory and antioxidant [21] (correlation of all compounds)
**Biflavonoids**		
Fukugetin	Fruit [43]	Analgesic [52], antioxidant [43]
Fukugiside	Fruit [43]	Analgesic [52], antioxidant [12]
morelloflavone-4′’’-*O*-β-d-glycoside	Fruit [43]	Antioxidant [12]
Amentoflavone	Leaf [41]	Anti-inflammatory and antioxidant [41]
Podocarpusflavone A	Leaf [41]	Anti-inflammatory and antioxidant [41]
**Benzophenones**		
Garcinol	Leaf [41]	Anti-inflammatory and antioxidant [41], anticancer, antiparasitic, action in nervous system [24]
7-epiclusianone	Leaf [22]/Fruit [47]	Antinociceptive and anti-inflamatory [22], antimicrobial [47], anticarcinogenic [49], leishmanicidal [21], schistosomicidal [50]
**Organic Acid**		
Galic acid	Leaf [41]	Anti-inflammatory and antioxidant [41]
**Flavonoid**		
Procyanidine	Leaf [41]	Anti-inflammatory and antioxidant [41]
**Xanthones**		
Guttiferone-A	Seeds [47]/Fruits [21]	Antimicrobial [47], photoprotective, and photochemopreventive [20] Leishmanicidal [21]
1,3,6,7-tetrahydroxyxanthone	Fruit [43]	Antioxidant [43]

**Table 2:** List of compounds presented in different parts of *Garcinia gardneriana* and related activities.

*Garcinia Gardneriana*		
Biflavonoids		
Compounds	Plant Part	Activity
GB-2a	Leaf [64], branches [59]	Antiedematogenic [64], anti-inflammatory [64], anticancer [65]
Gb-2a-7-*O*-glucoside	Branches [59]	Anticancer [65]
Volkensiflavone	Leaf [52]	Analgesic [52]
Fukugentin	Leaf [52]	Analgesic [52], anti-inflammatory [59], antioxidant [43]
Fukugiside	Leaf [52]	Analgesic [52], antioxidant [52]
GB-2a-II-4′-OMe	Leaf [52]	Analgesic [52]
**Flavonoid**		
**Compound**	**Plant Part**	**Activity**
Epicatechin	Leaf [58]	Antibacterial [58]
**Phytosterols**		
**Compound**	**Plant Part**	**Activity**
Sitosterol	Fruits [52]	Anti-inflammatory and anticancer [31]
stigmasterol	Fruits [52]	Anti-inflammatory and anticancer [31]
**Benzophenones**		
7-epiclusanone	Peel [52]	Antinociceptive and anti-inflammatory [22], antimicrobial [47], anticarcinogenic [49], leishmanicidal [21], schistossomicidal [50]
**Sesquiterpenes**		
α-copene	Peel [52]	-
α-muurolene	Peel [52]	-
γ-cadinene	Peel [52]	-
Cadinene	Peel [52]	-
**Triterpene**		
Oleanolic acid	Peel [52]	-

**Table 3:** List of compounds present in different parts of *Garcinia pedunculata* and related activities.

*Garcinia Pedunculata*		
Xanthones		
Compounds	Plant Part	Activity
1,3,6,7-tetrahydroxyxanthone	Heartwood [19]	Antioxidant [43]
1,3,5,7-tetrahydroxyxanthone	Heartwood [19]	Inhibits oxidation of LDL-c [45]
1,5-dihydroxy-3-methoxy-6′,6′-dimethyl-2*H*-pyrano(2′,3′:6,7)-4-(3-methylbut-2-enyl)xanthone	Peel [69]	-
1,5-dihydroxy-3-methoxy-4-(3-methylbut-2-enyl)xanthone	Peel [69]	-
Dulxanthone A	Peel [69]	-
Garbogiol	Peel [69]	Inhibition of α-glucosidade [10]
Pedunxanthone-A	Peel [69]	-
Pedunxanthone-B	Peel [69]	-
Pedunxanthone-C	Peel [69]	-
Pedunxanthone-D	Pericarp [33]	Anticancer [65]
Pedunxanthone-E	Pericarp [33]	Anticancer [65]
Pedunxanthone-F	Pericarp [33]	Anticancer [65]
1,6-dihydroxy-7-methoxy-8-(3-methyl-2-butenyl)-6′,6′-dimethylpyrane-(2′,3′:3,2)-xanthone	Pericarp [33]	-
6-*O*-demethyloliverixanthone	Pericarp [33]	-
Fuscaxanthone A	Pericarp [33]	Cytotoxic [16]
Cowanin	Pericarp [33]	Antimalarial [65]
Norcowanin	Pericarp [33]	Antiplasmodic [65]
Cowanol	Pericarp [33]	Antimalarial [65]
α-mangostin	Pericarp [33]	-
Mangostanol	Pericarp [33]	-
3-isomangostin	Pericarp [33]	-
1,7-dihydroxyxanthone	Pericarp [33]	-
**Benzophenones**		
Pedunculol	Dry fruits [70]	Antioxidant [16]
Isogarcinol	Dry fruits [70]	Anticancer, anti-inflammatory, antiparasitic, action in nervous system [24]
Garcinol	Dry fruits [70]	Anticancer, anti-inflammatory, antiparasitic, action in nervous system [24]
2,4,6,3′,5′-pentahydroxybenzophenone	Heartwood [19]	-
**Biflavonoids**		
GB-1a	Heartwood [19]	Antioxidant [84]
volkensiflavone	Heartwood [19]	Antitumoral [74]
**Triterpene**		
Oleanolic acid	Peel [69]	-

**Table 4:** List of compounds presented in different parts of *Garcinia cambogia* and their related activities.

*Garcinia Cambogia*		
Xanthones		
Compounds	Plant Part	Activity
Garbogiol	Roots [85]	Inhibition of α-glucosid [10]
Rheedia xanthone A	Peel [85]	-
Oxy-guttiferone i	Fruits [85]	-
Oxy-guttiferone k	Fruits [85]	-
Oxy-guttiferone k2	Fruits [85]	-
Oxy-guttiferone m	Fruits [85]	-
**Benzophenones**		
garcinol	Peel [85]	Anticancer, anti-inflammatory, antiparasitic, action on nervous system [24]
isogarcinol	Peel [85]	Anticancer, anti-inflammatory, antiparasitic, action on nervous system [24]
Guttiferone i	Fruits [85]	-
Guttiferone n	Fruits [85]	-
Guttiferone j	Fruits [85]	-
Guttiferone k	Fruits [85]	Topoisomerase II inhibitor [87]
Guttiferone m	Fruits [85]	Topoisomerase II inhibitor [87]
**Organic Acids**		
Heterocyclic amines	Fruits [85]	Antiobesity [137]
Tartaric acid	Fruits [85]	-
Citric acid	Fruits [85]	-
Malic acid	Fruits [85]	Antimicrobial [138]
Garcinialactone	Fruits [85]	-

**Table 5:** List of compounds presented in different parts of *Garcinia mangostana* and their related activities.

Garcinia Mangostana		
Xanthones		
Compounds	Plant Part	Activity
α-Mangostin	Pericarp, whole fruit, stem, arils, and seed [159]	Antibacterial, antifungal, antihistamine, antiobesity, anticancer [159], neuroprotective, antineoplastic [134], antioxidant [168]
β-Mangostin	Pericarp, whole fruit, stem [159]	Antiparasitic, hypoglycemic, antiobesity [134], antioxidant [168]
γ-Mangostin	Pericarp, whole fruit [159]	Antibacterial, anti-inflammatory, antihistamine, anticancer, hepatoprotective [159], antineoplastic, hypoglycemic, antiobesity, neuroprotective [134]
(16E)-1,6-Dihydroxy-8-(3-hydroxy-3-methylbut-1-enyl)-3,7-dimethoxy-2-(3-methylbut-2-enyl)-xanthone	Not stated [159]	-
(16E)-1-Hydroxy-8-(3-hydroxy-3-methylbut-1-enyl)-3,6,7-trimethoxy-2-(3methylbut-2-enyl)-xanthone	Whole fruit [159]	-
1,2-Dihydro-1,8,10-trihydroxy-2-(2-hydroxypropan-2-yl)-9-(3-methylbut-2-enyl)furo [3,2-a]xanthen-11-one	Heartwood [159]	-
1,3,6,7-Tetrahydroxy-xanthone	Pericarp [159]	-
1,3,6,7-Tetrahydroxy-2,8-(3-methyl-2-butenyl)-xanthone-P1	Pericarp [159]	-
1,3,6-Trihydroxy-7-methoxy-2,8-(3-methyl-2-butenyl)-xanthone-P2	Leaves [159]	-
1,3,8-Trihydroxy-4-methyl-2,7-diisoprenylxanthone	Heartwood [159]	-
1,3,7-Trihydroxy-2,8-di-(3-methylbut-2-enyl)-xanthon	Leaves [159], Pericarp [169]	-
1,3-Dihydroxy-2-(2-hydroxy-3-methylbut-3-enyl)-6,7-dimethoxy-8-(3-methylbut-2-enyl)-xanthone	Heartwood [159]	-
1,5-Dihydroxy-2-(3-methylbut-2-enyl)-3-methoxy-xanthone	Heartwood, stem [159]	-
1,5-dihydroxy-2-isopentyl-3-methoxy xanthone	Heartwood [159]	-
1,5,8-Trihydroxy-3-methoxy-2-(3-methylbut-2-enyl) xanthone	Heartwood [159], Pericarp [159]	-
1,6-Dihydroxy-2-(2-hydroxy-3-methylbut-3-enyl)-3,7-dimethoxy-8-(3-methylbut-2-enyl)-xanthone	Pericarp [159]	-
1,6-Dihydroxy-3-methoxy-2-(3-methyl-2-buthenyl)-xanthone	Pericarp [159]	-
1,6-Dihydroxy-3,7-dimethoxy-2-(3-methylbut-2-enyl)-8-(2-oxo-3-methylbut-3-enyl)-xanthone	Whole fruit [159]	-
1,6-Dihydroxy-3,7-dimethoxy-2-(3-methylbut-2-enyl)-xanthone	Heartwood [159]	-
1,6-Dihydroxy-8-(2-hydroxy-3-methylbut-3-enyl)-3,7-dimethoxy-2-(3-methylbut-2-enyl)-xanthone	Heartwood [159]	-
1,7-Dihydroxy-2-(3-methylbut-2-enyl)-3-methoxy-xanthone	Pericarp [159]	-
1,7-dihydroxy-2-isopentyl-3-methoxy xanthone	Pericarp [159]	-
11-Hydroxy-1-isomangostin	Not stated [159]	-
1-Hydroxy-2-(2-hydroxy-3-methylbut-3-enyl)-3,6,7-trimethoxy-8-(3-methylbut-2-enyl)-xanthone	Heartwood [159]	-
1-Isomangostin	Pericarp [159]	-
1-Isomangostin hydrate	Pericarp [159]	-
2-(γ,γ-Dimethylallyl)-1,7-dihydroxy-3-methoxyxanthone	Pericarp, arils [159]	-
2,3,6,8-Tetrahydroxy-1-isoprenylxanthone	Not stated [159]	-
2,8-bis-(γ,γ-Dimethyallyl)-1,3,7-trihydroxyxanthone	Arils [159]	-
3-Isomangostin	Pericarp [159]	Hypoglycemic, antiobesity, neuroprotective [134]
3-Isomangostin hydrate	Pericarp [159]	-
5,9-Dihydroxy-8-methoxy-2,2-dimethyl-7-(3-methylbut-2-enyl)-2H,6Hpyrano-(3,2,6)-xanthene-6-one	Fruit hull [159]	-
6-Deoxy-7-demethylmangostanin	Whole fruit [159]	
6-methoxy–bis pyrano xanthone	Pericarp [159]	Antioxidant [170]
6-*O*-Methylmangostanin	Not stated [159]	
7-*O*-Demethyl mangostanin	Pericarp [159]	Anticancer [169]
8-Deoxygartanin	Pericarp, whole fruit [159]	-
8-Hydroxycudraxanthone	Pericarp [159]	-
9-hydroxycalabaxanthone	Bark [171]	Neuroprotective [134]
BR-Xanthone-A	Pericarp [159]	-
BR-Xanthone B	Pericarp [159]	-
Calabaxanthone	Arils [159]	-
Cratoxyxanthone	Pericarp, stem, whole fruit [169]	-
Cudraxanthone	Pericarp [159]	-
Demethylcalabaxanthone	Whole fruit, arils, seed [159]	Antibacterial [159]
Dulxanthone-A	Bark [171]	Antibacterial [171]
Garcimangosone A	Fruit hull [159]	-
Garcimangosone B	Pericarp [159]	-
Garcimangosone C	Pericarp [159]	-
Garciniafuran	Heartwood [159]	Neuroprotective [134]
Garcinone B	Pericarp, whole fruit [159]	-
Garcinone C	Whole fruit [159]	Neuroprotective [134]
Garcinone D	Pericarp, whole fruit, stem [159]	Antibacterial [161], neuroprotective [134], antioxidant [47]
Garcinone E	Pericarp, whole fruit [159]	-
Garcinoxanthone-A	Not stated [134]	Antinociceptive, anti-inflammatory [134]
Garcinoxanthone-B	Not stated [134]	Antinociceptive, anti-inflammatory [134]
Garcinoxanthone-C	Not stated [134]	Antioxidant [46], antinociceptive, anti-inflammatory [134]
Garcinoxanthone-d	Not stated [134]	Antinociceptive, anti-inflammatory [134]
Garcinoxanthone-E	Not stated [134]	Antinociceptive, anti-inflammatory [138], antibacterial [171]
Garcinoxanthone-F	Not stated [134]	Antinociceptive, anti-inflammatory [134]
Garcinoxanthone-G	Not stated [134]	Antinociceptive, anti-inflammatory [134]
Garmoxanthone	Bark [171]	Antibacterial [171]
Gartanin	Pericarp, whole fruit [159]	Antineoplastic, hypoglycemic, antiobesity, neuroprotective [134], antioxidant [170]
Isogarcinol	Not stated [134]	Antinociceptive, anti-inflammatory [134], antibacterial [43]
Mangosharin	Stem [159]	-
Mangostaxanthone-I	Pericarp [133]	α-amylase inhibitor [136]
Mangostaxanthone-II	Pericarp [133]	α-amylase inhibitor [136]
Mangostaxanthone-III	Pericarp [168]	AGE* inhibitor, antioxidant [168]
Mangostaxanthone-IV	Fruits [172] Pericarp [164]	AGE* inhibitor, antioxidant [168]
Mangostaxanthone-V	Fruits [172]	-
Mangostaxanthone-VI	Fruits [172]	-
Mangostaxanthone-VII	Pericarp [136]	-
Mangostanaxanthone-VIIII	Pericarp [136]	α-Amylase inhibitory [136]
Mangostanate	Pericarp [172]	-
GlucosidaMangostanin	Pericarp [159]	Antibacterial [159]
Mangostanol	Wholefruit, stem [159]	-
Mangostenol	Pericarp [159]	-
Mangostenone A	Pericarp [159]	-
Mangostenone B	Pericarp [159]	-
MangostenoneC	Whole fruit [159]	Hypoglycemic, antiobesity [134]
Mangostenone D	Whole fruit [159]	Hypoglycemic, antiobesity [134]
Mangostenone E	Whole fruit [159]	
Mangostenone F	Not stated [134]	α-glucosidase inhibitor, antineoplastic [134]
Mangostinone	Pericarp, whole fruit [159]	-
Nigrolineaxanthone T	Bark [171]	-
Nor-mangostin	Fruits [172]	-
Rubraxantone	Pericarp [168]	Antioxidant [168]
Smeathxanthone A	Pericarp [159]	-
Thwaitesixanthone	Whole fruit [159]	-
Tovophyllin A	Pericarp [159]	-
Tovophyllin B	Pericarp [159]	-
Toxyloxanthone A (trapezifolixanthone)	Pericarp [159]	-
trapezifolixanthone	Pericarp [169]	-
1,7-dihydroxyxanthone	Pericarp [159]	-
Euxanthone	Pericarp [159]	-
Caloxanthone A	Pericarp [159]	-
Macluraxanthone	Pericarp [159]	-
Mangostingone [7-methoxy-2-(3-isoprenyl)-8-(3-methyl-2-oxo-3-buthenyl)-1,3,6-trihydroxyxanthone	Pericarp [159]	-
**Benzophenones**		
2,4,6,3′,5′-pentahydroxybenzophenone		
Garcimangosone D	Pericarp [159]	-
Maclurin	Pericarp, heartwood [159]	-
maclurin-6-*O*-β-d-glucopyranoside	Pericarp [134]	Hypoglycemic, antiobesity [134]
Kolanone	Pericarp [159]	-
**Anthocyanidins**		
Chrysanthemin	Pericarp [159]	-
Cyanidin-3-*O*-glucoside	Not stated [159]	-
**Biflavonoid**		
proanthocyanidin A2	Pericarp [173]	Anti-HIV [174]
**Flavonoid**		
Epicatehin	Pericarp [159]	Antidiabetic, antioxidant [173], hypoglycemic, antiobesity [134]
Aromadendrin-8-C-β-d-glucopyranoside	Pericarp [134]	Hypoglycemic, antiobesity [134]
**Megastigmanesulphoglycoside**		
4-*O*-sulpho-β-d-glucopyranosylabscisate	Pericarp [173]	Antioxidant [173]
